# Perceived functional resilience in schools according to key stakeholders

**DOI:** 10.1038/s41598-023-50892-w

**Published:** 2024-01-10

**Authors:** Arielle Kaim, Maya Siman-Tov, Shahar Lev-Ari, Bruria Adini

**Affiliations:** 1 Department of Emergency and Disaster Management, School of Public Health, Sackler Faculty of Medicine, Tel Aviv University, P.O. Box 39040, 6139001 Tel Aviv, Israel; 2 Sheba Medical Center, The Gertner Institute for Epidemiology and Health Policy Research, 5266202 Tel Hashomer, Ramat-Gan, Israel; 3 ResWell Research Collaboration, School of Public Health, Tel Aviv University, P.O. Box 39040, 6139001 Tel Aviv, Israel; 4 Department of Health Promotion, School of Public Health, Sackler Faculty of Medicine, Tel Aviv University, 6139001 Tel Aviv, Israel

**Keywords:** Psychology and behaviour, Sustainability

## Abstract

Amid the COVID-19 outbreak, Israel and numerous other governments closed schools as a precaution, leading to a sudden shift to online learning. The aim of the current study is to provide foundational insight into the perceived readiness of the school system to withstand future adversities, based on the challenges, complexities, as well as successes in adaptation faced by stakeholders during COVID-19. In this cross-sectional study, we assess the perceived levels of functional resilience of the school system among the key stakeholders of the Israeli education system-high school students, parents, teachers, and principals, as well as a composite functional resilience scale. The composite functional resilience consists of 10 main indexes: communication during distance learning (DL) and frontal learning (FL); Perceived stress scale-4 (PSS); psychosocial aspects during distance learning (DL) and frontal learning (FL); digital literacy; pedagogic support; resources; infrastructure; and distance versus frontal learning. The study findings demonstrate differences according to the stakeholders with regard to the perceived functional resilience and the composite functional resilience scores (e.g., students with respect to both of these scores exhibit the lowest results, while teachers display the highest scores). Furthermore, no one variable was significant across the board for all stakeholders in predicting the perceived functional resilience, with the most common predictors among the stakeholders being digital literacy, pedagogic support, PSS, as well as communication during distance and frontal learning. The findings of this study reveal areas for recommended priority actions to be conducted among school system stakeholders.

## Introduction

During the COVID-19 pandemic, school closures were a widespread response among governments, with over 100 countries, including Israel, implementing this measure to contain the spread of the virus^1^. This resulted in a sudden shift to online education and virtual instruction, as schools were ordered to stop in-person classes for most students^2–4^. The pandemic's trajectory has caused fluctuations in measures, with new lockdowns and the emergence of more contagious variants leading to further school closures and remote learning. According to UNESCO, this has affected over 90% of the world's schoolchildren, approximately 1.6 billion students (2020). By June 2021, some countries had experienced school closures for up to 60 weeks^5^. The World Bank estimated that a 5-month school shutdown could result in a loss of $10 trillion in terms of educational progress^6,7^. The most disadvantaged children, who often depend on school for education, nutrition, and health needs, faced the harshest impacts of the temporary school closures^8^. During the COVID-19 crisis in the United States, access to quality education, technology, and the internet varied widely. Both students from rural and urban regions struggled to connect to the internet, with as many as one-third of urban students unable to attend online classes^9^. This resulted in a significant portion of schoolchildren being excluded not only from learning but also from social interaction with their peers.

School closures, however, have affected society as a whole, not just students. School staff and administration, including teachers and principals, have experienced significant impacts^10,11^. Teachers and administrators had to adapt to online teaching and faced challenges, including a lack of prior experience with online teaching, increased workload, and isolation from students and colleagues^12–15^. With schools being a source of safeguarding and supervision for children, the closures have resulted in parents having to stay home, with inevitable economic consequences, or leaving children unsupervised^16^. Furthermore, the shift from frontal (in-class) to online learning has put parents in an educational role at home^17^.

The concept of resilience has gained significant attention during the COVID-19 pandemic as researchers have explored resilience at the individual, community, national, organizational, and systemic levels^18–22^. Masten^23^ defines resilience as a system’s ability to adapt to disturbances that threaten its survival or development. Recent research has focused on operationalizing resilience and evaluating it. One emerging focus within resilience research is functional resilience, which is defined as a system’s ability to resist, absorb, and respond to shocks while maintaining its critical functions, and then recover or adapt^24,25^. There is limited research on functional resilience in the education system, but literature from the hospital system suggests that factors such as the vulnerability of structural and non-structural components, critical infrastructure, impacts on facility occupants, the role of external stakeholders, and policies to absorb and respond to adverse impacts play a role in the functional resilience^26^. The systemic level requires engagement and assessment of stakeholder involvement to align goals and objectives^27–29^. Findings from the context of the hospital system support the interdependence between perceptions of resilience among the key systemic players and the multilevel/ multistage nature of an organization’s resilience^30^. The literature suggests a need for further investigation using the functional resilience model at the systemic or institutional level, and the development of a comprehensive set of metrics and indicators to assess functional resilience.

The COVID-19 pandemic and its resulting school closures have exposed the vulnerability of education systems worldwide^31–33^. The sudden disruption and subsequent integration of the school system and its stakeholders highlight the need to ensure the functional resilience of schools in both face-to-face and remote learning environments. To assess the potential for change and improvement in the education system, it is crucial to understand the factors that affect its functional resilience. The pandemic provides an opportunity for educational change, but without benchmarks, it is difficult to determine if these changes will lead to desired outcomes for schools in Israel and globally^34^. Determining the functional resilience of a system as a whole can be done through the creation of comprehensive metrics, but it’s also important to consider the varying perspectives of multiple stakeholders. Their views on what the system’s main purpose and key functions are and how well the system is achieving these functions can provide valuable insights. To improve the functional resilience of the system, it's essential to understand the perspectives and perceptions of all stakeholders and integrate their feedback in shaping relevant interventions to improve system-wide functional resilience.

This study aims to evaluate the perceived level of functional resilience among key stakeholders in the school system. This will provide foundational insight into the perceived readiness of the school system to withstand future adversities, based on the challenges, complexities, as well as successes in adaptation faced by stakeholders during the COVID-19 pandemic.

## Methods

### Study design

Considering the importance of achieving an understanding of the perceived resilience of the school system according to the various stakeholders, a cross-sectional study was conducted in October–November 2022, two and a half years after the initial move to distance learning. The total sample included 1802 participants, distributed among the four key stakeholders of the education system: 10th–12th grade students (N = 1000), parents (N = 301), teachers (N = 449), and principals (N = 52) among 890 Israeli Jewish high schools. To participate in the study, those recruited had to confirm their willingness to participate voluntarily in the study. The data was gathered by iPanel, the largest Israeli internet panel company which consists of over 140,000 panelists representing all demographic and geographic sectors (http://www.ipanel.co.il). The internet panel provides an online platform that adheres to the stringent standards of the European Society for Opinion and Marketing Research (ESOMAR). In line with the ethical committee approval, parental/ legal guardian consent was obtained for the minor participants for study participation.

### The study tools

The four questionnaires were tailored to each of the stakeholders’ groups and consisted of: 1) a composite functional resilience scale that consists of 10 main indexes: communication during distance learning (DL); communication during frontal learning (FL); Perceived stress scale-4; psychosocial aspects during distance learning (DL); psychosocial aspects during frontal learning (FL); digital literacy; pedagogic support; resources; infrastructure; and distance versus frontal learning, as well as 2); a perceived functional resilience scale (See Kaim et al.^35^), based on items and indices that were developed specifically for this study, except for the perceived stress scale (PSS), which was based on a validated tool (PSS-4)^36^. The number of items was not identical for the four tools, as to reflect the relevant relationships between stakeholders/roles and functions that each stakeholder plays. The four questionnaires were validated by twenty content experts and pilot-tested on 25 individuals prior to their dissemination. All items were assessed on a 5-point Likert scale) ranging from 1 = Disagree to a very great extent to 5 = Agree to a very great extent, with the exception of PSS-4 measured on 1 = never to 5 = Very often. The reliability of the composite functional resilience scale was measured by Alpha Cronbach and for each of the four stakeholders were (α = 0.628) among students, (α = 0.649) among parents, (α = 0.796) among teachers, and (α = 0.720) among principals. The perceived functional resilience was measured by 13 items among students and parents and 19 items among teachers and principals. The components of this index encompass attitudes towards functioning through the distance learning phase during the COVID-19 crisis, the ability of schools to take away lessons learned and adapt them from the COVID-19 school closures, and preparedness for future adversities. Thirteen of the questions were identical (though adapted to each specific population). The reliability of the scale was measured by Alpha Cronbach and results for each of the four stakeholders were (α = 0.925) among students, (α = 0.726) among parents, (α = 0.949) among teachers, and (α = 0.944) among principals.

### Statistical analysis

Descriptive statistics were used to describe the participants’ demographic characteristics (frequency, mean, and standard deviation) of all four stakeholders. In addition, descriptive statistics were used to describe the characteristics of the schools sampled (%), and to determine the spread tendency and central tendency in the perceived and composite functional resilience indexes. A one-way ANOVA test was used to assess variability between stakeholders. A post hoc test (Bonferroni) was further conducted to enable the identification of the differences among the varied groups for two of the indexes. A general linear model was conducted to assess for statistically significant differences between perceived functional resilience and the composite functional resilience score among the four stakeholders. Linear regressions were performed to determine the factors affecting the perceived functional resilience of the school system after negating multi-collinearity and homoscedasticity check. Only the variables that were significant in the one-way analysis were entered into the regression model.

All statistical analyses were performed using SPSS software version 28. P-values lower than 0.05 were considered to be statistically significant.

### Ethical approval

The study was conducted in accordance with the Declaration of Helsinki, and approved by the Institutional Review Board (or Ethics Committee) of Tel Aviv University (number 0004549–1 from February 13th, 2022) and the Ministry of Education (number 12379 from April 28th, 2022). 

### Informed consent

Informed consent was obtained from all subjects involved in the study.

## Results

### Respondents and school sample characteristics

The study sample consisted of 1802 participants, including 1000 students, 301 parents, 449 teachers, and 52 principals. Of the student population, 52.5% are male, the average age is 16.7 ± 0.8, and 54.4% are secular. Among the parent sample population, 68.4% are female, the average age is 48.0 ± 5.1, 59.1% are secular, 85.4% are in a relationship with children, 44.6% have a bachelor’s degree, and 37.6 indicate above average level of income earnings. Among the teachers’ sample population, 80.8% are female, the average age is 41.6 ± 11.8, 43% are secular, 71.7% are in a relationship with children, 50.2% have a bachelor’s degree, and 44.9% indicate below average level of income earnings. Among the principals’ sample population, 55.8% are female, the average age is 46.7 ± 8.9, 53.8% are secular, 88.6% are in a relationship with children, 66.7% have a master’s degree and above, and 56.3 indicate above average level of income earnings.

**Figure 1:**
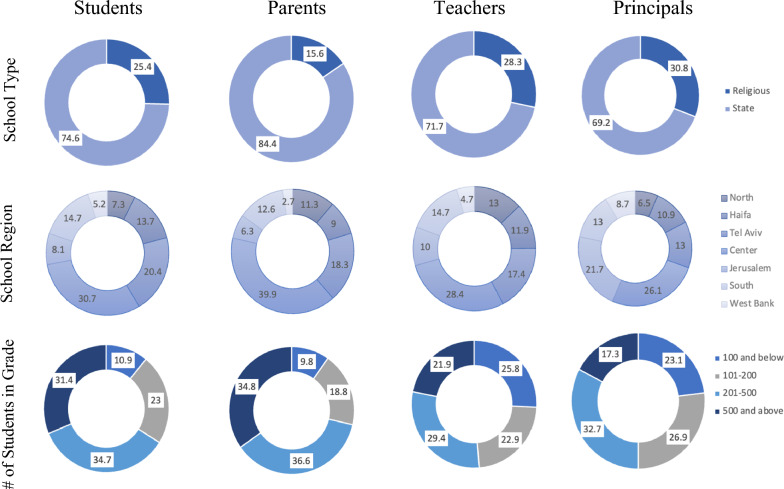
School characteristics (in %) according to the four stakeholder groups (students, parents, teachers and principals). *Note:* Missing data from sample regarding school characteristics: type of school = 0; school region = 38; number of students in school grade = 319. See Kaim et al.^35^, for further reference to school characteristics.

A total of 890 Jewish schools within Israel were sampled, with 67.1% of the sampled schools being state schools and the largest school region representation being the center (29.6%); 33.5% of the sample schools had between 201 and 500 enrolled students. Of the stakeholders, the majority of students (74.6%), parents (84.4%)/ teachers (71.7%) / and principals (69.2%) sampled came from state schools. The core academic program in Religious Schools mirrors the State Schools' curriculum, but Religious Schools also place a strong focus on intensive Jewish and religious education. Both types of schools have comparable levels of resource availability. The distribution of the stakeholders according to the characteristics of the school is presented in Fig. 1.

### Mean levels of perceived functional resilience and the composite functional resilience index

**Figure 2:**
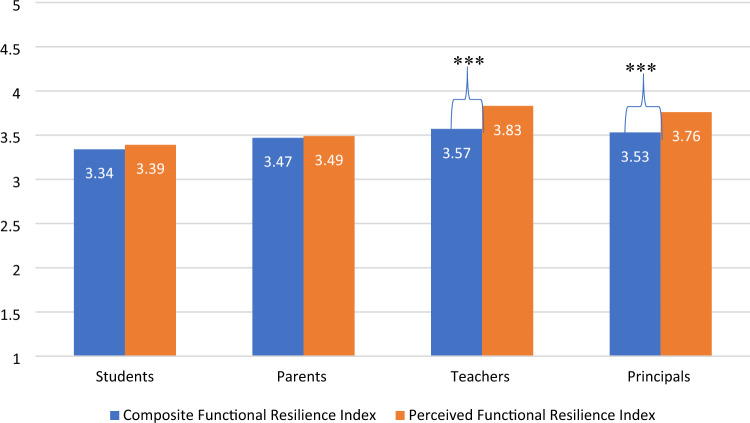
Mean composite functional resilience index versus mean perceived functional resilience according to four stakeholders (students, parents, teachers, principals). *Note:* According to Bonferroni multiple comparisons test, for perceived functional resilience, significant differences were observed between students and teachers (*p* < 0.001), students and principals (*p* < 0.001), and parents and teachers (*p* < 0.05); while for the composite functional resilience Index, significant differences were found between students and parents (*p* < 0.001), students and teachers (*p* < 0.001); parents and teachers(*p* < 0.05). A general linear model was conducted to assess for statistically significant differences between perceived functional resilience and the composite functional resilience score among the four stakeholders. Significance of *p* < 0.001) is denoted with ***.

Differences by stakeholders (students, parents, teachers, and principals) with respect to the composite functional resilience score and the perceived functional resilience score are displayed in Fig. 2. The mean perceived functional resilience index scores were 3.39 ± 0.76 for students, 3.49 ± 0.86 for parents, 3.83 ± 0.72 among teachers, and 3.76 ± 0.70 among principals, while the composite score respectfully were 3.35 ± 0.50, 3.48 ± 0.47, 3.59 ± 0.52, and 3.55 ± 0.47. Repeated measures ANOVA model was conducted to assess for statistically significant differences between perceived functional resilience and the composite functional resilience score among the four stakeholders. Statistically significant differences were found between the two measurements (F = 36.79, *p* < 0.001) as well as an interaction effect between the four groups (F = 10.17, *p* < 0.001). In the pairwise comparison post-hoc test in each group, no significant differences between the perceived functional resilience score and the composite functional resilience score were found among students and parents, however among teachers and principals, a statistically significant difference was found (*p* < 0.05).

Furthermore, the trends regarding the lowest mean scores are consistently among students for both the perceived and composite functional resilience scores, while the highest mean scores of both indices are found among teachers. The correlation between the two indexes is 0.682 for the whole sample together.

### Predictors of the perceived functional resilience among each of the stakeholders

Linear regression analysis was performed to predict the variables that impact on perceived functional resilience of each stakeholder group (Table 1: students; Table 2: parents; Table 3: Teachers; Table 4: Principals). The variables integrated into the model of each respective stakeholder are listed in each table. The variables entered into the model predict 58.61% of the variance of the dependent variable among students. The significant variables from highest to lowest predictive values are: digital literacy (B = 0.210), communication DL (B = 0.207), perceived stress scale (B = 0.139), type of school (B = 0.131), communication CL (B = 0.121), pedagogic support (B = 0.118), psychosocial aspects CL (B = 0.101), and distance versus frontal learning (B = 0.088).

**Table 1:** Results of linear regression for predicting perceived functional resilience among students.

Variables	Coefficient B	Beta coefficient B	T value	Significance value
Gender (1-male, 2-female)	− 1.849E−5 0.000	0.000	− 0.001	1.000
Type of school (1-state school 2-religious school)	0.131	0.075	3.619	**< 0.001**
Communication—distance learning (DL)	0.207	0.254	8.915	**< 0.001**
Communication-frontal learning (FL)	0.121	0.149	5.948	**< 0.001**
*Perceived stress scale (PSS)	0.139	0.131	5.625	**< 0.001**
Psychosocial aspects—distance learning (DL)	0.003	0.005	0.182	0.856
Psychosocial aspects—frontal learning (FL)	0.101	0.113	4.682	**< 0.001**
Digital literacy	0.210	0.255	9.145	**< 0.001**
Pedagogic support	0.118	0.149	5.317	**< 0.001**
Infrastructure	− 0.007	− 0.008	− 0.337	0.736
Distant versus frontal learning	0.088	0.111	4.006	**< 0.001**

Significant values are in [bold].

R^2^ = 0.586, F = 126.799, sig < 0.001.

**Table 2:** Results of linear regression for predicting perceived functional resilience among parents.

Variables	Coefficient B	Beta coefficient B	T value	Significance value
Type of school (1-state school 2-religious school)	0.218	0.092	2.324	**0.021**
Communication—distance learning (DL)	0.054	0.038	0.588	0.557
Communication—frontal learning (FL)	0.281	0.266	5.698	** < 0.001**
*Perceived stress scale (PSS)	0.136	0.108	2.501	**0.013**
Psychosocial aspects—distance learning (DL)	0.066	0.080	1.803	0.072
Psychosocial aspects—frontal learning (FL)	0.081	0.080	1.785	0.075
Digital literacy	0.245	0.294	5.226	**< 0.001**
pedagogic support	0.171	− 0.210	4.042	**< 0.001**
Infrastructure	− 0.100	− 0.080	− 1.308	0.192
distant versus frontal learning	0.148	0.151	3.031	**0.003**

Significant values are in [bold].

R^2^ = 0.591, F = 41.915, sig < 0.001.

**Table 3:** Results of linear regression for predicting perceived functional resilience among teachers.

Variables	Coefficient B	Beta coefficient B	T value	Significance value
Type of school (1-state school 2-religious school)	0.014	0.009	0.243	0.808
Relationship with children	− 0.085	− 0.053	− 0.911	0.363
No relationship, no children	− 0.28	− 0.012	− 0.228	0.819
No relationship, yes children	− 0.052	− 0.022	− 0.437	0.662
Communication—distance learning (DL)	0.189	0.201	3.780	**< 0.001**
Communication—frontal learning (FL)	0.242	0.229	4.881	**< 0.001**
*Perceived stress scale (PSS)	0.151	0.146	3.678	**< 0.001**
Psychosocial aspects—distance learning (DL)	− 0.043	− 0.049	− 0.937	0.349
Psychosocial aspects—frontal learning (FL)	0.098	0.087	1.965	**0.050**
Digital literacy	0.155	0.157	2.630	**0.009**
Pedagogic support	0.178	0.254	5.136	**< 0.001**
Infrastructure	− 0.055	0.073	− 1.698	0.090
Distant versus frontal learning	0.019	0.025	0.527	0.598

Significant values are in [bold].

R^2^ = 0.453, F = 27.712, sig < 0.001.

**Table 4:** Results of linear regression for predicting perceived functional resilience among principals.

Variables	Coefficient B	Beta coefficient B	T value	Significance value
Communication—distance learning (DL)	0.407	0.385	3.075	**0.004**
Communication—frontal learning (FL)	0.095	0.102	0.653	0.517
*Perceived stress scale (PSS)	− 0.031	− 0.032	− 0.354	0.725
Psychosocial aspects—frontal learning (FL)	0.319	0.517	3.125	**0.003**
Digital literacy	0.012	0.011	− 0.093	0.927
Infrastructure	− 0.090	− 0.116	− 1.194	0.239

Significant values are in [bold].

R^2^ = 0.741, F = 21.483, sig < 0.001.

The results of the regression model for predicting the perceived functional resilience among parents are presented in Table 2. The variables entered into the model predict 59.1% of the variance of the dependent variable. The significant variables from highest to lowest predictive values are: communication CL (B = 0.281), digital literacy (B = 0.245), type of school (B = 0.218), pedagogic support (B = 0.171), digital versus frontal learning (B = 0.148), perceived stress scale (B = 0.136), and psychosocial aspects DL (B = 0.066).

The results of the regression model for predicting the perceived functional school among teachers are presented in Table 3. The variables entered into the model predict 45.3% of the variance of the dependent variable. The significant variables from highest to lowest predictive values are: communication CL (B = 0.242), communication DL (B = 0.189), pedagogic support (B = 0.178), digital literacy (B = 0.154), and the perceived stress scale (B = 0.151),

The results of the regression model for predicting the perceived functional resilience among principals are presented in Table 4. The variables entered into the model predicted 74.1% of the variance of the dependent variable. Only two variables were found to be significant including communication DL (B = 0.407) and psychosocial aspects CL (B = 0.319).

## Discussion

Understanding the key stakeholders’ perceptions of the school systems response during the COVID-19 pandemic and the ability of the school system to maintain functional continuity of services, as well as the adoption of lessons learned and preparedness for future crises is a crucial component of identifying where the response and resilience of the system can be improved^26^.

The findings of this investigation demonstrate several interesting phenomena. The first are the differences according to the stakeholders with regard to the perceived functional resilience and the composite functional resilience scores. The composite functional resilience score was developed to assess variability among schools (further described in Kaim et al.^35^), however the findings reveal an interesting variability among the stakeholders as well. Students with respect to both of these scores exhibit the lowest results, while teachers display the highest scores. This may suggest that students were hit hardest among the key stakeholders, and thus are more critical concerning the resilience of the school system, a finding supported by previous literature^37,38^. In contrast, teachers and principals presented the highest levels in both perceived and composite functional resilience, which may indicate that even though they were not fully prepared for the exceptional challenges posed by COVID-19, they were able to come up with innovative solutions and effectively adjust their practices^39^. Contrary to previous findings which indicate that organizations tend to underestimate their capacities to function following disruptions^40,41^, here we see that teachers and principals show significantly higher perceived levels as compared to the actual composite functional resilience score. It may be that the school staff (both teachers and principals) were under great pressure to display effective performance during the pandemic, where they reflect on the school’s function as a proxy of their own performance^42^. Hence, there is variability revealed between the perceived versus the composite functional resilience score, as well as between senior managers and staff members with those receiving the services (parents and students)^40^. The findings indicate the need to ensure that all key stakeholders are actively engaged in crisis preparedness and response, as well as in resilience initiatives.

No one variable was significant across the board for all four stakeholders in predicting the perceived functional resilience. The most common predictors of perceived functional resilience among 3 of the four stakeholders were digital literacy, pedagogic support, PSS, as well as communication during distance and frontal learning. Digital literacy for example, was a salient factor among students, parents, and teachers, while not among principals, potentially reflecting the requirements for the nature of the roles that the three stakeholders took on throughout the pandemic, and the tools necessary to perform these roles, as compared to the principals. Teachers had to navigate teaching through digital platforms, while students and parents had to learn or train themselves to teach, respectfully via digital platforms. Similarly, among the same three stakeholders, pedagogic support highly predicted the perceived functional resilience, may reflect the high need for support among the three stakeholders, that encountered the greatest adjustments as compared to how they were executing their roles during times of normalcy. For example, parents needed assistance in supplementary teaching/ guidance academically of their children, teachers needed to adapt new methodology and materials for teaching, while students needed to adapt the modality in which they learn and study. Furthermore, the negative predictive association between PSS and higher functional resilience among students, parents and teachers is a finding highly supported by the literature in the context of other forms of resilience (individual, community, and national)^43,44^. The PSS-4 measurement assesses attitudes and feelings in the past month, indicative of longer-term repercussions on distress. In the context of the study, the negative relationship between distress and perceived functional resilience aligns. Lastly, communication during frontal learning among the three stakeholders above played a significant role in predicting the perceived functional resilience. This may be reflective of the maintenance of high levels of communication beyond the school closure disruptions, which were perceived as positive for the functionality of the school system (for example, among parents and teachers, where it has been noted in the literature that communication increased during the pandemic^45,46^.

On the individual stakeholder levels, the most salient factors predicting perceived functional resilience in the context of students were digital literacy, communication during distance learning, as well as the type of school (state versus religious schools). The finding that learning in a religious school versus a state school better predicts perceived functional resilience is in line with former studies. A higher level of religiosity has previously been associated with higher levels of resilience^47,48^. Digital literacy played the most important role in predicting functional resilience among students reflecting the important role of digital literacy education^49,50^. This emphasizes the need for increased institutional investment in students and faculty training in digital literacy readiness, as being equipped with the necessary skillsets would contribute to improved functional resilience in the context of future adversities.

Among parents, the most significant factors predicting functional resilience were the type of school their child belongs to, communication during frontal learning, as well as digital literacy. Here once again, the findings suggest that if the parents’ child learns in a religious school, this predicted higher perceived functional resilience by the parents. Furthermore, the higher the level of digital literacy as well as communication during frontal learning, the higher the perceived functional resilience. It has been noted that parents throughout the pandemic were visibly more involved in the schooling process, and the relationships improved with teachers as well^45,46^. The higher perceived functional resilience indicates that potentially the higher-level involvement continued following the reopening of school doors, reflecting higher perceived resilience among parents.

Among teachers, pedagogic support as well as communication during distance and frontal learning were the leading factors in predicting the functional resilience. Effective communication plays a crucial role in organizational resilience^51^. Regarding pedagogic support, it equips teachers with the resources and training necessary to successfully navigate challenges and foster a favorable learning environment^52–54^. This support can include professional development opportunities, access to instructional materials, and ongoing coaching and mentorship where with enhanced capacity from pedagogical support, teachers are better equipped to manage adversities.

Lastly, among principals the main factors predicting perceived functional resilience are communication during distance learning and psychosocial aspects (frontal learning), returning to the importance of prioritizing communication. Principals can foster a culture of resilience, ensuring that teachers and students have the support they need to succeed in the face of challenges. Intricately tied to communication are psychosocial aspects, where communication fosters a sense of belonging and improved morale among those involved^55^. Furthermore, among principals, the results of the linear regression model displayed a very high level of prediction (74.1%), which may suggest the higher homogeneity of the principals’ population and their respective attitudes.

The study has several pertinent limitations. The first limitation is the nature of utilizing an online panel for response collection. While this methodology allows for rapid turnover of information and provides a representative sample of the youth and adult Israeli population, the study conclusions are limited to individuals who have access to an internet source and high levels of computing skills. Furthermore, the cross-sectional design of the study bases the attitude findings concerning distance learning on memory, as they were not assessed during schools’ closure, but rather only after.

## Conclusions

The findings of this study reveal areas for recommended priority actions to be conducted among school system stakeholders. Institutional policy-level intervention and support to the more vulnerable stakeholders are fundamental to the overall future resilience of school systems in the context of future crises. Particularly for example, as educational environments evolve, the benefits of digital literacy become increasingly significant, potentially bolstering the functional resilience of the entire educational community by enhancing adaptive skills and access to digital resources. To the best of our knowledge, very limited studies have been carried out to evaluate the factors that play the most salient role in perceived functional resilience among four key stakeholders of the education system. In light of this, comprehensive assessments of functional resilience—considering both the composite and perceived capacities—should be conducted over time to identify variabilities in response to diverse adversities. Additionally, to foster a more robust understanding, we recommend that assessments of functional resilience (both composite and perceived) be conducted over time to identify variabilities according to a variety of adversities and occurrences. Furthermore, the study should be expanded to additional school types with additional characteristics evaluated, such as Jewish versus Arab schools in the Israeli context, private versus public schools, etc.

## Data Availability

The data collected in this study is not available in any public repository due to the regulations of the funder. The analyzed data will be made available to requesting researchers upon a reasonable request to the corresponding author.
